# The Pathology and Surgical Treatment of Tumors

**Published:** 1901-06

**Authors:** 


**The Pathology and Surgical Treatment of Tumors.** By N. Senn, M.D. Second Edition. Pp. 718. London and Philadelphia: W. B. Saunders. 1900.

Owing to the progress of knowledge, successive generations view the problems presented by the pathology and treatment of disease, from different standpoints; hence every age needs its own interpreters.

This is specially true of the vast subject of tumour formation, with which Dr. Senn has dealt so ably in the volume now before us. His book on the pathology and treatment of tumours is in many respects an excellent one; and it enjoys the distinction of being the only modern work of the kind extant.

We should like to have seen, however, the essentially modern features of the subject more prominently and thoroughly set forth than we find them, for there is apparent a bias towards mediævalism and the doctrines of the days of other years.

Those who write on subjects still in their infancy must of necessity deal largely in vague generalities, for the very good reason that particularities are then scanty or wanting; hence these pioneers are obliged to classify their subject matter under broad and comprehensive headings, which conveniently cloak the scantiness of the data at their disposal.

In treating of tumours, such was the method adopted by the authors of the first half of the nineteenth century—Müller, Virchow, Broca, etc.—who described the different kinds of tumours under such comprehensive headings as cancer, sarcoma, lipoma, myoma, cystoma, etc.

Under the totally different conditions now prevailing, it seems questionable whether Dr. Senn has done well to follow the "old guard" in this respect.

The chief characteristic of modern tumour science is the immense accumulation of detailed facts, relating to the life-history of the different *local* varieties of the disease, that has taken place during the last half-century. We want to see these *membra disjecta* co-ordinated and arranged, in a concise and accurate manner, so that their significance may be readily grasped by the ordinary reader. In this respect, it seems to us that Dr. Senn's work leaves much to be desired.

It has been said of Austerlitz that it was a battle lost before it was begun, owing to the faulty initial dispositions of the allies. In like manner, it may be said of Dr. Senn's book that it has to a large extent failed to attain the end in view, owing to its facts being marshalled in accordance with the ancient order of ideas. The new wine ill accords with the old bottles. Thus the work, although young of years, savours of the nineteenth, rather than of the twentieth, century.

Whether we view the subject from the genetical, morphological, pathological, clinical, or therapeutic standpoints, there can be no doubt that, in a modern work, tumours should be described in accordance with the organs and localities whence they originate, rather than in vague general terms, which convey to the mind only confused ideas, instead of that *netteté* which is so desirable.

Another important respect in which Dr. Senn seems to have missed the modern spirit of investigation is in his neglect of the numerical method, which has of late rendered such immense service to medical science. Owing to this lapse, his descriptions often lack accuracy of statement, as well as symmetry of development.

For instance, what can be more inaccurate than the statement made on page 67: "that, on the whole, the male sex is more predisposed to tumour-formation than is the female"? The truth is exactly the converse: thus we have found the ratio to be as follows—cancer, 5,017 females to 2,861 males; sarcoma 648 females to 702 males; non-malignant tumours, 3,434 females to 1,179 males; and cysts, 1,640 females to 449 males.

Again, on page 71, to show the relative frequency with which the different organs are affected with tumours, an ancient table by C. O. Weber is cited, which is most misleading. Yet plenty of thoroughly reliable modern statistics bearing on this important subject are available.

Owing to neglect of the numerical method, Dr. Senn has fallen into the serious mistake of maintaining that non-malignant tumours are specially prone to become malignant (p. 81). This relic of mediævalism can be shown to be totally false by the intelligent use of modern statistics; and we are surprised to find it reappearing in Dr. Senn's work, for it is a fetish to which many valuable lives have been unnecessarily sacrificed.

To show the prevalence of cancer in England, the Registrar-General's figures for the years 1860–70 are quoted (p. 203). We cannot help wondering why statistics thirty years out of date should be cited when recent ones are available, especially as these ancient data are now absolutely useless for the purpose in view, owing to the disease having since increased so rapidly.

On page 238 we find a statement to this effect: "One of the most instructive evidences of the influence of prolonged irritation and inflammation in the causation of carcinoma is chronic eczema of the nipple, known as 'Paget's disease of the nipple.'" Here, again, an acquaintance with the numerical method would have prevented Dr. Senn from making a statement than can be proved to be without any foundation. The real truth of the matter we have found to be as follows: When Paget said that chronic eczema of the nipple was "very often" followed by the development of cancer in the breast, there can be no doubt that he made a mistake. Paget adduced no statistical data in support of his opinion; but, since his publication, the subject has been numerically investigated on a large scale, with the result that cancer of the breast has been shown to follow eczema of the nipple *in only about ½ per cent. of the total cases.* We may, therefore, in spite of Dr. Senn's dictum, disabuse our minds of the idea that "Paget's disease" plays an important part in the pathogeny of mammary cancer. Eczematous affections of the nipple, etc., are very seldom followed by the outbreak of cancer in the breast, hence the mamma should never be extirpated for such affections, unless signs of concomitant malignant disease are also present.

